# Lactic acid bacteria in cow raw milk for cheese production: Which and how many?

**DOI:** 10.3389/fmicb.2022.1092224

**Published:** 2023-01-12

**Authors:** Luca Bettera, Alessia Levante, Elena Bancalari, Benedetta Bottari, Monica Gatti

**Affiliations:** Department of Food and Drug, University of Parma, Parma, Italy

**Keywords:** raw milk cheese, raw milk microbiota, cheese microbiota, cheese ripening, LAB, NSLAB, *Lacticaseibacillus*

## Abstract

Lactic Acid Bacteria (LAB) exert a fundamental activity in cheese production, as starter LAB in curd acidification, or non-starter LAB (NSLAB) during ripening, in particular in flavor formation. NSLAB originate from the farm and dairy environment, becoming natural contaminants of raw milk where they are present in very low concentrations. Afterward, throughout the different cheesemaking processes, they withstand chemical and physical stresses becoming dominant in ripened cheeses. However, despite a great body of knowledge is available in the literature about NSLAB effect on cheese ripening, the investigations regarding their presence and abundance in raw milk are still poor. With the aim to answer the initial question: “which and how many LAB are present in cow raw milk used for cheese production?,” this review has been divided in two main parts. The first one gives an overview of LAB presence in the complex microbiota of raw milk through the meta-analysis of recent taxonomic studies. In the second part, we present a collection of data about LAB quantification in raw milk by culture-dependent analysis, retrieved through a systematic review. Essentially, the revision of data obtained by plate counts on selective agar media showed an average higher concentration of coccoid LAB than lactobacilli, which was found to be consistent with meta-taxonomic analysis. The advantages of the impedometric technique applied to the quantification of LAB in raw milk were also briefly discussed with a focus on the statistical significance of the obtainable data. Furthermore, this approach was also found to be more accurate in highlighting that microorganisms other than LAB are the major component of raw milk. Nevertheless, the variability of the results observed in the studies based on the same counting methodology, highlights that different sampling methods, as well as the “history” of milk before analysis, are variables of great importance that need to be considered in raw milk analysis.

## 1. Introduction

Microbiological and biochemical changes in the curd are crucial factors for the production of raw-milk, ripened cheeses. The microbial ecology of this cheese variety consists of a complex interaction between starter lactic acid bacteria (SLAB, usually deliberately added for curd acidification) and non-starter LAB (NSLAB, adventitious milk contaminants from farm and dairy environments) from milking to ripening (Gatti et al., 2014; Blaya et al., 2018).

NSLAB frequently recovered from cheese are facultative heterofermentative lactobacilli (Beresford et al., 2001). *Lacticaseibacillus* [formerly *Lactobacillus casei* group, (Zheng et al., 2020)] is one of the most prevalent genera found in hard cooked, long-ripened cheeses. It includes the species *Lacticaseibacillus casei*, *Lacticaseibacillus paracasei*, and *Lacticaseibacillus rhamnosus*, which are of particular interest because of their proven role in cheese flavor formation during ripening (Bottari et al., 2018), as well as their potential health benefits through the consumption of fermented foods (Hill et al., 2018; De Filippis et al., 2020). Their trend from low abundance in raw milk, to dominance in ripened cheese, involves adaptation to chemical and physical stresses throughout the cheesemaking and ripening processes (i.e., heat-related, acidic, osmotic, and oxidative stresses), besides their ability to grow using energy sources other than lactose (Gatti et al., 2014). These are the physiological criteria used for the delineation of the name of the new genus *Lacticaseibacillus* from *caseus* (cheese; Zheng et al., 2020).

The mechanisms responsible for NSLAB survival to stresses encountered during cheesemaking might be attributed to various strategies of adaptation, such as the capability to utilize different energy sources (Lazzi et al., 2014; Papadimitriou et al., 2016) or the activation of strategies that can increase their tolerance to the food manufacturing process, such as the recently described toxin-antitoxin systems (Levante et al., 2019, 2021). Although NSLAB have been extensively studied for their role in cheese ripening (De Dea Lindner et al., 2008; Solieri et al., 2012; Sgarbi et al., 2013, 2014; Levante et al., 2017; Bottari et al., 2020), and reviews are available on this topic (Beresford et al., 2001; Settanni and Moschetti, 2010; Montel et al., 2014; Gobbetti et al., 2015; Bottari et al., 2018), the source of their origin and their relative abundance in raw milk are not well clarified yet.

Further research is necessary to demonstrate whether live microorganisms are present in milk inside a healthy mammary gland (Oikonomou et al., 2020), although milk endogenous bacterial contamination *via* enteromammary pathway has been hypothesized (Addis et al., 2016). However, it is known that microbial colonization occurs from different sources throughout the route from farm to cheese factory (Gobbetti et al., 2018) and raw milk represents an ideal environment for the growth of many microorganisms (Quigley et al., 2013). To shed light on which and how many of these biotypes will be part of the ripened cheese microbiota, it is of primary importance to know their initial abundance in raw milk, since the number of bacterial cells is known to be one of the most important factors in determining microbial activity in food (Fleet, 1999; Giraffa, 2004). It is expected that in the next future quantitative methods such as flow cytometry will help understanding how microbial loads of certain species in raw milk may affect the microbiome of resulting cheeses (Skeie et al., 2019; Porcellato et al., 2021), as it was also shown for other complex microbiomes (Vandeputte et al., 2017).

Although many studies deal with the description of the complex dairy ecosystem focusing on LAB in raw milk, often reporting isolation, identification, and relative quantification of the most abundant species, they rarely provide absolute quantification. With the aim to answer the initial question: “which and how many lactic acid bacteria are present in cow raw milk used for cheese production?,” this review analyses and comments on the data of the studies available in the literature addressing LAB quantification in raw milk used for the production of different cow’s milk cheeses.

The different analytical techniques used for LAB evaluation in milk can be divided into two methodological approaches: (I) conventional culture-dependent methods which analyze microorganisms after their growth in liquid or solid media; (II) more recent culture-independent methods which directly detect microbial nucleic acids, avoiding the culturing step. A prior careful consideration of numerous factors (e.g., taxonomic resolution, workload) is necessary to define the appropriate methodology for a certain purpose (Temmerman et al., 2004). These different approaches are complementary, thus their combination in a polyphasic approach is suggested to set up more complete and accurate studies (Ercolini et al., 2001; Delbès et al., 2007; Agrimonti et al., 2019) that better describe the dynamic evolution of microbial communities in food ecosystems, in particular in the complex transformation of raw milk to ripened cheese.

In both methods, the qualitative information (i.e., microorganisms taxonomic identification) can be addressed by common molecular analysis (such as PCR-and sequencing-based methods). On the other hand, the quantitative results differ: most of the culture-independent studies on LAB in raw milk report the relative abundance (%) of prevalent taxa; culture-dependent studies mainly based on plate counts, report instead results as Colony Forming Units (CFU/mL).

For this reason, data collected from the studies applying different approaches have been discussed separately in the following two chapters: the first one aims at giving an overview of LAB’s abundance within the complex microbiota of cow raw milk through the review of recent taxonomic studies; the second includes a collection of data about quantification of LAB by culture-dependent analysis in cow raw milk.

## 2. Culture-independent quantification

Culture-independent techniques available for milk and cheese microbiota analysis are constantly evolving and have been extensively reviewed elsewhere (Randazzo et al., 2009; Ndoye et al., 2011; Quigley et al., 2011; Neviani et al., 2013; Addis et al., 2016; Levante et al., 2020; Tilocca et al., 2020). Common outlines of these reviews are: (I) the usefulness of the culture-independent approach in describing complex ecosystems such as raw milk and cheese, and the ability to overcome culture-dependent limits (e.g., ability to distinguish between dead or viable cells by a combination of DNA-and RNA-based approaches), although a polyphasic approach is recommended; (II) -omics approaches are expected to greatly contribute in shedding light on microbial ecology dynamics.

Methods for absolute quantification at the species level have been implemented, such as Fluorescence *in-situ* Hybridization [FISH (Bottari et al., 2006)], quantitative PCR [qPCR (Agrimonti et al., 2019)], and Total Bacterial Count computed with the percentage of taxon after 16S rRNA Amplicon Sequencing (Props et al., 2017; Skeie et al., 2019; Porcellato et al., 2021). However, to the authors’ knowledge, no studies applied these methods for the absolute quantification of the *Lacticaseibacillus* group in cow raw milk used for cheese production, except for Masoud and colleagues who quantified *Lacticaseibacillus rhamnosus* in raw milk used for Danish cheese production (Masoud et al., 2012).

Different studies are instead available in the literature about the characterization of raw milk microbiota by amplicon-based high-throughput sequencing (HTS) analysis. This method is successful to describe changes in microbiota related to seasonality, geographical origin, and the microbiota evolution at different steps of cheese making, but it suffers from bias because results are usually reported as relative abundance of taxa which are not converted to quantitative values (Skeie et al., 2019), and identification of bacteria beyond the genus level is often not possible (Claesson et al., 2010; Dreier et al., 2022). On the other hand, a fundamental advantage of these studies is the possibility to use the generated raw sequences to perform meta-analysis.

A very useful tool to conduct the meta-analysis is FoodMicrobionet [FMBN (Parente et al., 2016, 2019; De Filippis et al., 2018)], a collection of datasets created by 16S rRNA gene amplicon HTS studies of food bacterial communities. This tool was already used to widely review the microbiota of dairy milk, discussing comparisons between the pasture and feed, farm environments, teat skin, teat milk (from different species, also affected by diseases), bulk tank milk and finally also HTST (high-temperature short time) milk (Parente et al., 2020).

In the present review, the updated version of FMBN 4.1.2 (Parente et al., 2022), integrated with the taxonomy reclassification for the genus *Lactobacillus* (Zheng et al., 2020) and further recent studies on the raw milk microbiota, has been used. Among the studies available in the database on cow raw milk, those analyzing cow whole raw milk used for cheese production were selected (De Pasquale et al., 2014; Dolci et al., 2014; Calasso et al., 2016; De Filippis et al., 2016; Giello et al., 2017; Falardeau et al., 2019; Cremonesi et al., 2020; Kamimura et al., 2020; Nikoloudaki et al., 2021; Supplementary Tables 1, 2). This dataset included reads from 250 raw milk samples, classified in 1,594 total taxa belonging to 45 different phyla (*Chloroplast*, *Eukaryota*, *Mithocondria*, and unidentified OTUs at the domain level were removed). Nodes and edges tables (.gml file generated by FMBN Shiny app) were then imported into Gephi software [v 0.9.2 (Bastian et al., 2009)] for bipartite network analysis, i.e., network with nodes belonging to OTUs and samples.

**Figure 1:**
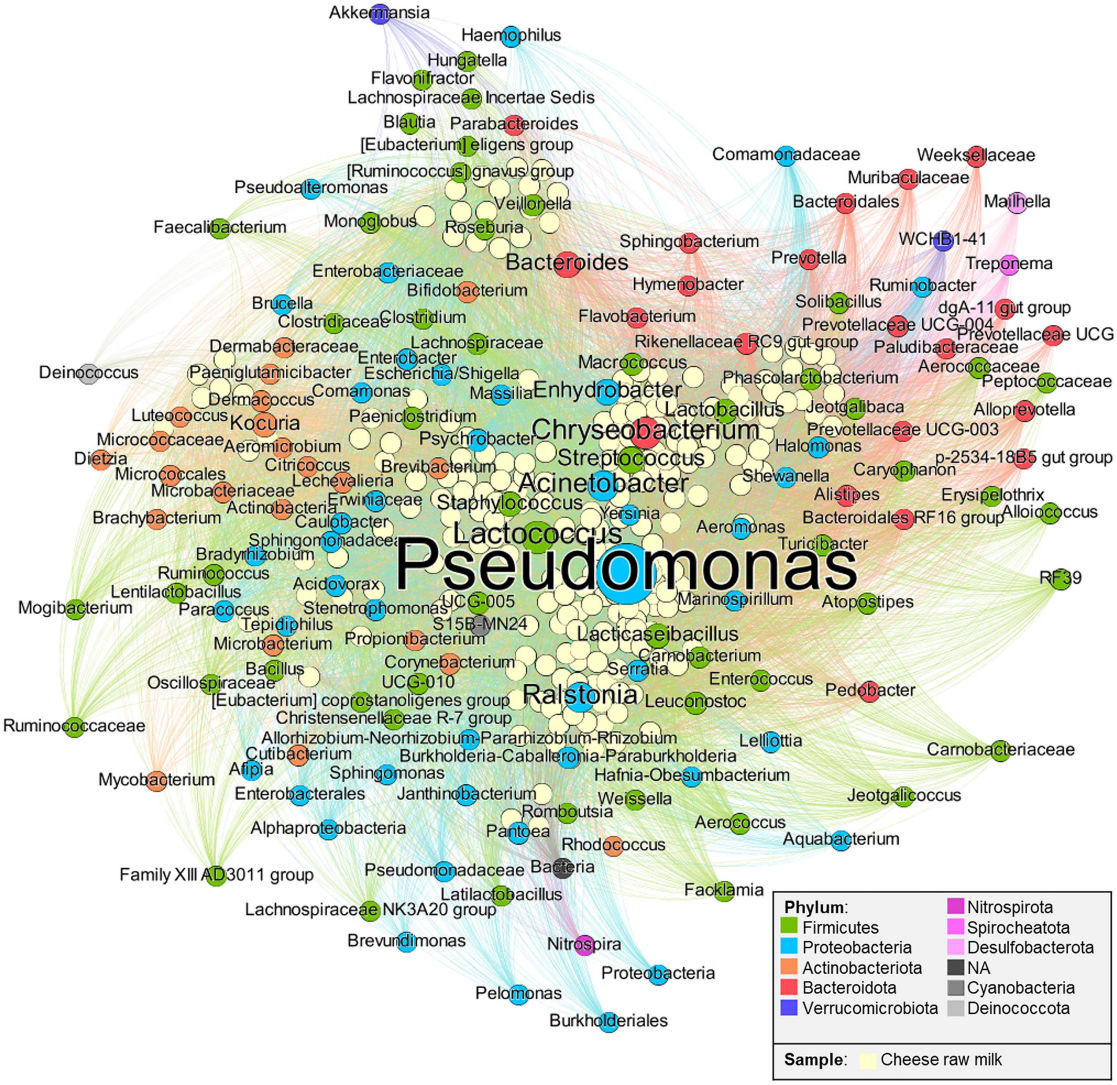
Bipartite network. Nodes represent cheese raw milk samples and Operational Taxonomic units (OTUs), linked to each other by edges. OTUs’ label and node size are directly proportional to their relative abundance in all the raw milk samples. Data were extracted from studies listed in Supplementary Table 2.

Nodes and edge statistics were calculated in Gephi: the *degree* is the number of connections to a certain node, meaning that for a sample, it represents the number of present OTUs, vice versa for an OTU, it represents the number of milk samples where it was found; the *weight* is a value assigned to an edge, corresponding to the OTU relative abundance in the sample; the *weighted degree* is the sum of *weights* for a node (Supplementary Table 3). Nodes and taxa label size are proportional to their *weighted degree*, hence the bigger they are, the more the OTU is abundant in raw milk. Only nodes with a *weighted degree* > 10 were considered for the network (Figure 1). ForceAtlas2 algorithm (Jacomy et al., 2014) was applied for the layout: as a consequence, taxa are closer to samples where they are present with a greater abundance (thus, taxa to the periphery are less abundant); also, taxa close to each other were present more frequently in the same samples.

After the filtering step, 149 taxa were retained (Supplementary Table 3), which belong to 10 different phyla. Part of the OTUs were classified only at the kingdom level (*Bacteria*, NA dark gray node). Among the most abundant genera, the psychrotrophic bacteria are present in significant proportions: these include *Pseudomonas* (23.85% of the tot *weighted degree* in the network), *Chryseobacterium* (6.56%), and *Acinetobacter* (6.01%). It is reasonable to assume that their dominance could be due to the refrigerating temperature usually applied for raw milk storage which decreases bacterial diversity (Raats et al., 2011).

According to these data, LAB relevant for cheese production are present as dominant taxa such as *Lactococcus* (7.24%), or subdominant such as *Streptococcus* (3.6%), *Lacticaseibacillus* (2.42%), *Lactobacillus* (2.35%), *Leuconostoc* (1.15%), and *Enterococcus* (0.41%). Despite their relatively low abundance in the raw milk ecosystem, these subdominant taxa can develop in subsequent cheese manufacturing. An example is found in long ripened cheeses, where the *Lacticaseibacillus* genus is known to become dominant throughout the ripening of raw milk, hard cooked, long-ripened cheeses (Bottari et al., 2018).

The low abundance of some subdominant species makes their isolation from raw milk difficult, limiting thus a potential targeted use. A strategy to overcome this limit could be the enrichment of the autochthonous raw milk microbiota through its spontaneous fermentation. Following this approach, Bancalari and colleagues carried out a spontaneous fermentation of Parmigiano Reggiano raw milk samples to isolate LAB strains potentially usable as adjunctive aromatic starters (Bancalari et al., 2017), while Galli and colleagues incubated the raw milk for 24 and 48 h, at 30°C and 40°C, with the purpose to isolate additional LAB with potentially high GABA (γ-aminobutyric acid) producing capabilities (Galli et al., 2022).

## 3. Culture-dependent quantification

### 3.1. A systematic review of plate counts on agar media of LAB in raw milk

#### 3.1.1. Search strategy

**Figure 2:**
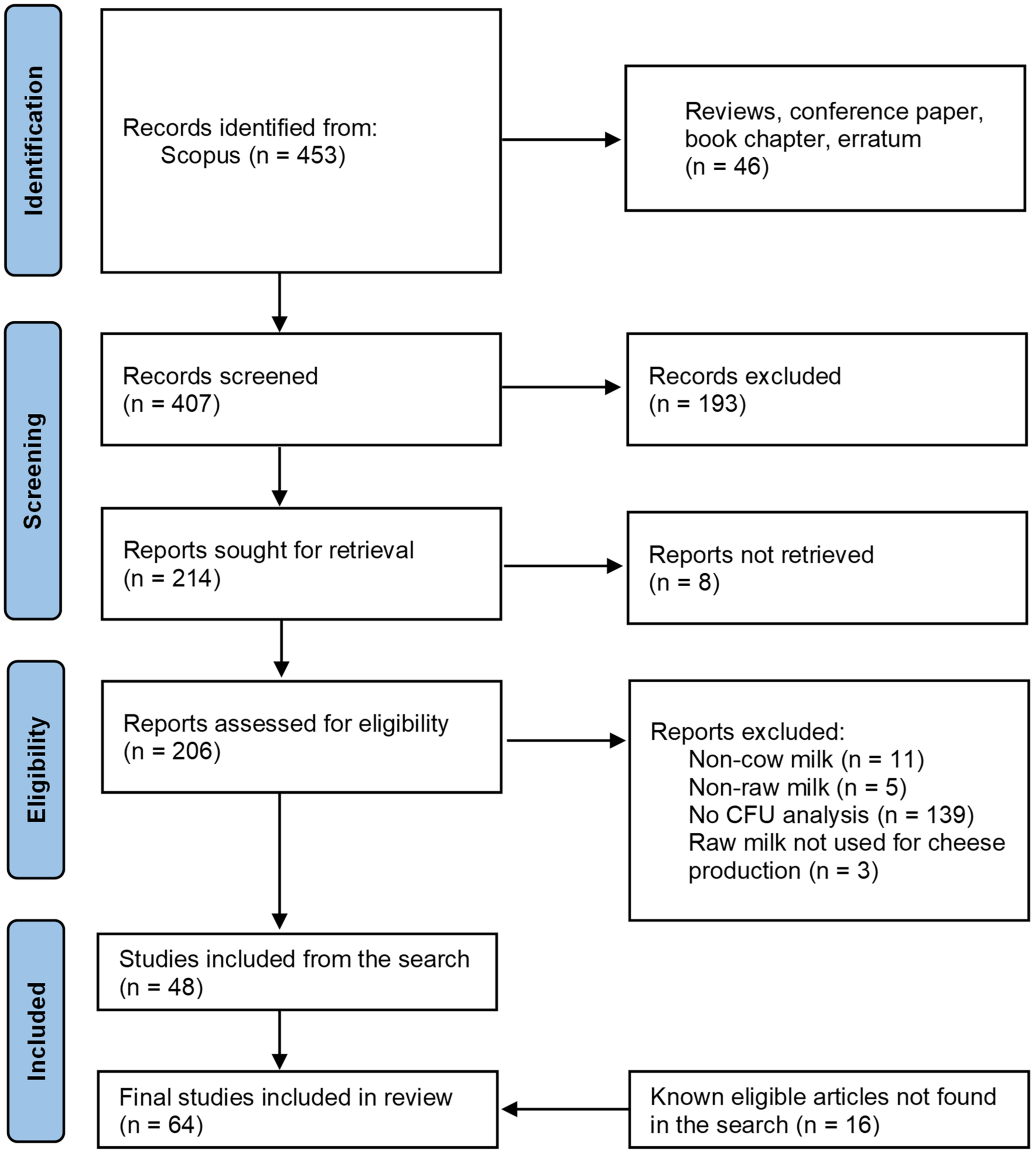
Flow diagram of the studies search for the systematic review of plate counts on agar media of Lactic Acid Bacteria in raw milk. CFU, Colony forming unit.

Articles were identified by searching in Scopus^1^ onthe10th of October 2022, using the following key words: TITLE-ABS-KEY ((({raw milk}) OR ({raw-milk}) OR ({bulk milk}) OR ({tank milk}) OR ({vat milk}) OR ({milk microbiota}) OR ({milk microflora}) OR ({Raw cows’ milk}) OR ({Raw milk/cheese})) AND (({lactic acid bacteria}) OR (lab) OR (lactobacilli) OR (lactobacillus) OR (lacticaseibacillus) OR (nslab)) AND (cheese) AND NOT (({human milk}) OR (camel) OR (goat) OR (ewe) OR (sheep) OR (deer) OR (donkey))). After a titles/abstract screening, articles were included in the review on the base of the following criteria about the milk analyzed: (1) it must be raw [as defined by (EC, 2004)]; (2) it must derive from cow; (3) it must be used for cheese production; (4) it must be analyzed for LAB Colony Forming Unit. In the Figure 2, the four-phase PRISMA flow diagram (Page et al., 2021) schematizing the studies search process is reported.

#### 3.1.2. Data elaboration

According to the definition of raw milk (EC, 2004), we considered only data from the analysis of milk samples which were not heat treated or undergone any treatment that has an equivalent effect. LAB quantification data (CFU/mL) evaluated by plate counts on elective or selective agar media after incubation at different temperatures were extracted and transformed in log_10_ scale when necessary. Quantification in broth media using the Most Probable Number (MPN) was rarely used (Mucchetti et al., 2009; Cremonesi et al., 2020). Results of standard total bacterial count through Plate Count Agar (PCA; EC, 2006; ISO 4833-2, 2013) have been also extracted when available. Data were grouped based on the LAB group analyzed, i.e., by growth media and incubation temperature used. Only the two largest, more robust datasets underwent a statistical analysis for the evaluation of a significant difference among the two LAB groups: (I) Lactobacilli, cultivated on Man-Rogosa-Sharpe (MRS) at 30–32°C (*n* = 82); (II) Coccoid LAB, cultivated on M17 at 30–32°C (*n* = 70). The two datasets (Supplementary Data 1) were checked for their normal distribution through the *Shapiro–Wilk* test; the homogeneity of variance was assessed with a *F-*test; finally, a *t-*test (*α* = 0.05) was applied. The data elaboration and statistical analysis were done in the R environment (R Core Team, 2022) using the packages “stats” and “tidyverse” (Wickham et al., 2019).

#### 3.1.3. Lactic acid bacteria concentration in raw milk for cheese production

Out of the 453 records retrieved from the systematic search (Figure 2), the first country in terms of publication number in this field is Italy (*n* = 102), followed by France (57), and Spain (48) (Scopus stats, data not shown). Together with Ireland, Switzerland, and Portugal, they represent the 59% of the articles retrieved by this search. This is evidence of the high research interest of the European Institutions in the dairy sector, reflecting the historic tradition of cheesemaking of the continent (Montel et al., 2014). The rest of the records were from Brazil, Turkey, Mexico and the United States of America.

Raw milk cheeses are produced worldwide. However, different cow raw milk cheeses analyzed in the studies retrieved through the literature search, were excluded from this review because the authors did not evaluated the LAB quantification in the raw milk by plate counts. Some examples are: Cotija [Mexico (Escobar-Zepeda et al., 2016)], Tulum [Turkey (Gezginc et al., 2022)], Fontina [Italy (Giannino et al., 2009)], Kraški and Tolminc [Slovenia (Trmčić et al., 2011)], Zlatar [Serbia (Terzic-Vidojevic et al., 2007)], Sir iz Mišine [Croatia (Vrdoljak et al., 2022)], Bitto [Italy (Colombo et al., 2009)], São Jorge [Portugal (Kongo et al., 2009)], and Poro de Tabasco [Mexico (De la Rosa-Alcaraz et al., 2020)].

**Figure 3:**
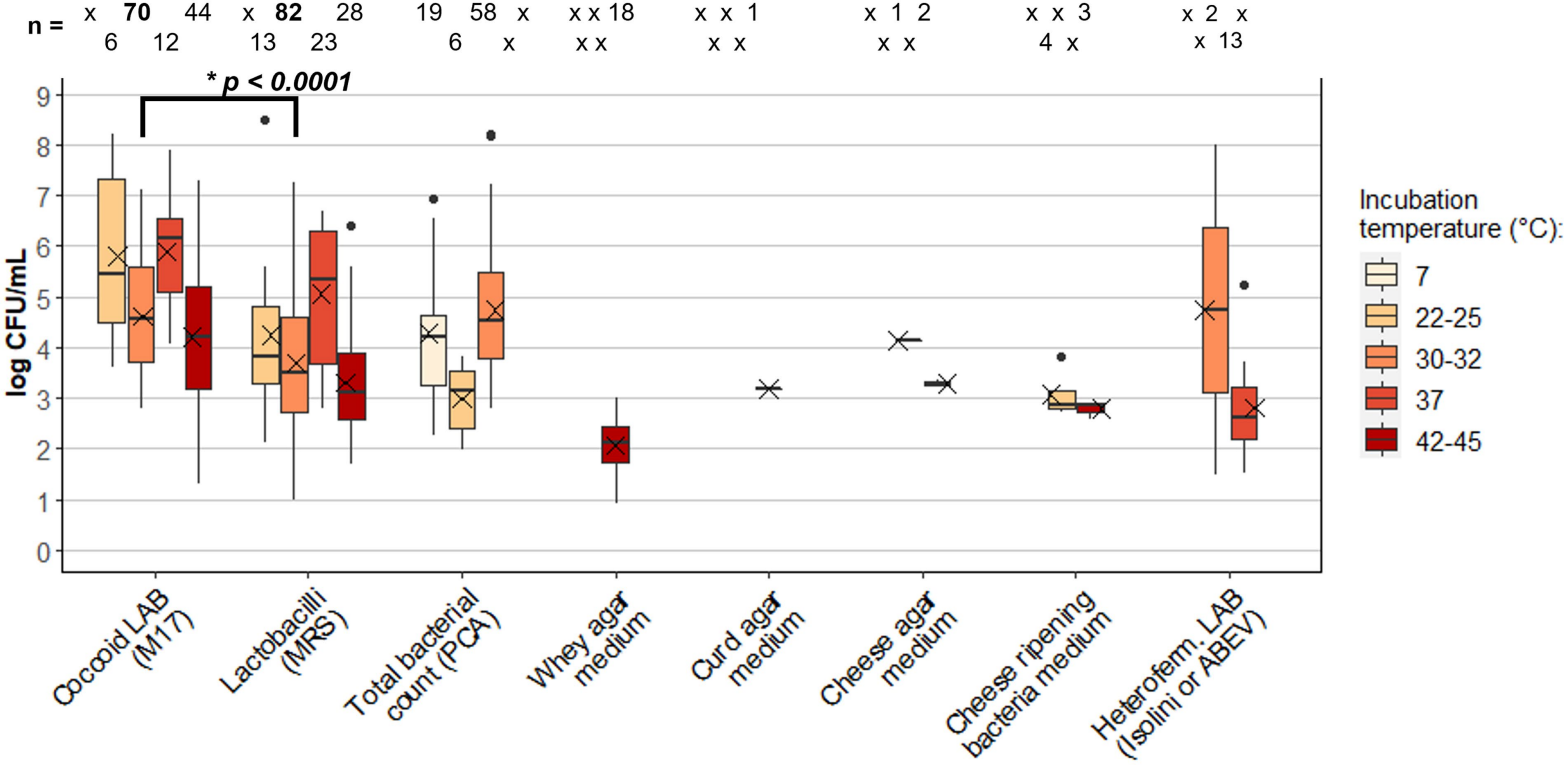
Box plot of Lactic Acid Bacteria (LAB) concentration (log CFU/mL) in cheese raw milk: comparison between different growth media and incubation temperatures. Extremes of the bars represent the max. and min. Values; the box lines indicate the 1st quartile, the median, and the 3rd quartile; the mean is indicated by the X within the box; isolated data points are outliers. The number of data (*n*) is reported at the top of each box (x = missing data). Data were extracted from studies listed in Table 1. The value of *p* (*p) resulting from the *t*-test applied to Coccoid LAB and Lactobacilli results incubated at 30–32°C is reported (datasets are reported in Supplementary Data 1). MRS, Man-Rogosa-Sharpe; PCA, plate count agar; ABEV, Arginine Bromocresol Meat Extract Vancomycin.

Table 1 reports the list of the 64 studies available in the literature that quantified LAB in cow raw milk used for the production of 42 types of cheese. Mean values of log CFU/mL have been grouped by media type and incubation temperature to define the core LAB raw milk microbiota (Figure 3). Thus, the observed variability is likely explained by the fact that other factors take part in the modulation of LAB load in raw milk, such as sampling procedures, milk pre-treatments (e.g., skimming), and seasonality. Furthermore, although the studies considered eligible in this review have in common the use of the same growth media and incubation temperature, they may have slight differences in the analysis procedure, such as plate incubation time, degree of aerobic/anaerobic conditions, use of selective additives (Supplementary Data 2).

**Table 1:** List of cheeses whose raw milk was analyzed for LAB concentration through culture-dependent techniques.

n.	Cheese	Country	Origin certification	Reference
1	Arzúa	Spain	–	Centeno et al. (1994)
2	Bergkäse	Austria	–	Eliskases-Lechner et al. (1999)
3	Caciocavallo di Castelfranco	Italy	PAT	Giello et al. (2017)
4	Caciocalvallo Palermitano	Italy	PAT	Settanni et al. (2012)
5	Caciocavallo Pugliese	Italy	–	Calasso et al. (2016)
6	Caciotta	Italy	–	Calasso et al. (2016)
7	Caciotta Montefeltro	Italy	–	Aquilanti et al. (2011)
8	Camembert de Normandie	France	PDO	Desmasures et al. (1997) and Henri-Dubernet et al. (2008)
9	Cantal	France	PDO	De Freitas et al. (2007)
10	Casín	Spain	PDO	Alegría et al. (2009)
11	Casizolu	Italy	PAT, Slow Food Presidia	Mangia et al. (2016)
12	Castelmagno	Italy	PDO	Dolci et al. (2008)
13	Cheddar	Ireland, United States	–	McSweeney et al. (1993), Rehman et al. (2000a,b, Gelsomino et al. (2001), Agarwal et al. (2006), and Hickey et al. (2007)
14	Cheese Basse-Normandie area	France	–	Denis et al. (2001) and Mallet et al. (2012)
15	Cheese Quebec area	Canada	–	Gagnon et al. (2020)
16	Cheese Savoie-Haute Savoie area	France	–	Michel et al. (2001)
17	Chihuahua	Mexico	–	Béjar-Lio et al. (2020)
18	Comté	France	PDO	Bouton et al. (1998)
19	Dil pasta-filata	Turkey	–	Irkin (2010)
20	Emmental de Savoie	France	PGI	Sohier et al. (2012)
21	Fior di Latte di Agerola	Italy	–	Coppola et al. (2006)
22	Genestoso	Spain	-	Arenas et al. (2004)
23	Grana Padano (including Trentingrana)	Italy	PDO	Santarelli et al. (2013), Bava et al. (2021), Franciosi et al. (2011a,b, 2012), Monfredini et al. (2012), Cremonesi et al. (2020)
24	Kashar	Turkey	–	Çetinkaya and Ece Soyutemiz (2006)
25	León	Spain	–	Rodriguez Medina et al. (1995)
26	Minas	Brasil	–	Castro et al. (2016) and Luiz et al. (2017)
27	Montasio	Italy	PDO	Carraro et al. (2011) and Marino et al. (2008)
28	Nite pasta-filata	Slovakia	–	Medvedová et al. (2020)
29	Nostrano di Primiero	Italy	PAT	Poznanski et al. (2004)
30	Pannerone	Italy	PAT	Mucchetti et al. (2009)
31	Parmigiano Reggiano	Italy	PDO	Coppola et al. (2000), Gatti et al. (2008), Neviani et al. (2009), Bortolazzo et al. (2010), Coloretti et al. (2016), and Franceschi et al. (2021)
32	Piedmont hard cheese	Italy	–	Bautista-Gallego et al. (2014)
33	Provolone del Monaco	Italy	PDO	Aponte et al. (2008)
34	Ragusano	Italy	PDO	Randazzo et al. (2002)
35	Saint-Nectaire	France	PDO	Delbès et al. (2007)
36	Salers	France	PDO	Callon et al. (2004)
37	San Simón	Spain	PDO	García Fontán et al. (2001)
38	Swiss-type	France	–	Beuvier et al. (1997)
39	Toma tipo Piemonte	Italy	–	Astegiano et al. (2014)
40	Traditional Mountain Cheese Trentino alpine area	Italy	–	Carafa et al. (2016, 2019, 2020), Franciosi et al. (2009)
41	Traditional Rugova	Kosovo	–	Ajazi et al. (2018)
42	UK blue-veined	United Kingdom	–	Yunita and Dodd (2018)

PDO, Protected Designation of Origin; PGI, Protected Geographical Indication (EP and Council of EU, 2012); PAT, Prodotti Agroalimentari Tradizionali (Mipaaf, 2022).

The total number of values found in literature about LAB quantification in raw milk used for Grana Padano cheese is 103, the highest among the values found for the other cheeses addressed in this review. This indicates a high research interest in this cheese type. The raw milk used for Grana Padano PDO (Protected designation of origin) cheese (including Trentingrana) was analyzed by sampling whole evening raw milk and skimmed morning raw milk, thus testing the effect of overnight creaming (Franciosi et al., 2011a; Santarelli et al., 2013). With a similar sampling procedure, also the microbial composition of morning whole raw milk and vat raw milk was evaluated (Monfredini et al., 2012), investigating also different storage temperatures (Franciosi et al., 2011b, 2012). More recently, the effect of the use of different machine cleaning detergents on the raw milk microbiota was investigated by sampling bulk tank raw milk at the farm and skimmed raw milk (Cremonesi et al., 2020), whereas Bava and colleagues analyzed bulk tank raw milk sampled at farms which had different management practices (Bava et al., 2021). In total, six studies quantified the LAB in raw milk used for Parmigiano Reggiano (Table 1), which allowed the collection of 28 values, the highest data number after Grana Padano and Traditional Mountain Cheese from Trentino alpine area (103 data in seven studies and 70 data in four studies, respectively). Raw milk used for Parmigiano Reggiano PDO is also skimmed by overnight creaming, and different studies analyzed evening whole and morning skimmed raw milk (CRPA, 2011; Coloretti et al., 2016). Franceschi and colleagues sampled bulk raw milk from tanks directly at the farm after storage at two different temperatures (Franceschi et al., 2021). Other studies analyzed vat raw milk, obtained by mixing whole evening raw milk and morning skimmed raw milk (Coppola et al., 2000; Gatti et al., 2008; Neviani et al., 2009; CRPA, 2011).

The vat raw milk has also been studied in several other types of cheese: Provolone del Monaco (Aponte et al., 2008), Castelmagno (Dolci et al., 2008), Saint-Nectaire (Delbès et al., 2007), Swiss-type (Beuvier et al., 1997), Traditional Mountain cheese (Carafa et al., 2016, 2019), Cheddar (Gelsomino et al., 2001), Arzúa (Centeno et al., 1994), Cantal (De Freitas et al., 2007) and Fior di Latte di Agerola (Coppola et al., 2006). In the case of Caciotta, Caciocavallo Pugliese, and Emmental de Savoie the raw milk was sampled from tanks at the cheese factory (Sohier et al., 2012; Calasso et al., 2016), while for other cheeses directly at the farm, sometimes evaluating the effect of seasonality (Desmasures et al., 1997; Arenas et al., 2004; Franciosi et al., 2009; Mallet et al., 2012; Castro et al., 2016; Luiz et al., 2017), breeding practices (Giello et al., 2017; Gagnon et al., 2020), or transhumance period (Carafa et al., 2020).

Many studies, instead, did not report detailed information about the raw milk samples analyzed (McSweeney et al., 1993; Rodriguez Medina et al., 1995; Rehman et al., 2000a,b; Denis et al., 2001; García Fontán et al., 2001; Randazzo et al., 2002; Agarwal et al., 2006; Çetinkaya and Ece Soyutemiz, 2006; Aquilanti et al., 2011; Carraro et al., 2011; Yunita and Dodd, 2018).

The main growth media used in the 64 reviewed studies were de Man-Rogosa-Sharpe (MRS; De Man et al., 1960) elective for lactobacilli (55 studies), M17 (Terzaghi and Sandine, 1975) elective for coccoid LAB (40 studies), and PCA (31 studies) for total bacterial count. The results of 5 growth temperature ranges are shown in Figure 3: 7°C for psychrotrophs; 22–25, 30–32 and 37°C for mesophiles; 42–45°C for thermophiles.

PCA medium was modified for coccoid LAB count (PCA-BCP, Supplementary Data 2), to analyze raw milk used to produce Camembert de Normandie [2.91 log CFU/mL raw milk in winter period; 2.84 log CFU/mL in spring/summer period (Desmasures et al., 1997)] and cheese of Basse Normandie area [1.91 log CFU/mL raw milk in winter period; 1.87 log CFU/mL in spring period (Mallet et al., 2012)]; for this reason, these values were not included in Figure 3. Since the average of total bacterial counts at 30–32°C (4.74 ± 1.26 log CFU/mL) is similar in comparison with LAB counts, especially for M17 counts, this may indicate that LAB represent the majority of mesophiles among culturable bacteria in raw milk. The slightly higher average concentration of total psychrotrophs in comparison with total mesophiles at 22–25°C is likely caused by the low number of data found in the literature for this temperature range (*n* = 19 and *n* = 6, respectively), in addition to the high values of 6.53 and 6.93 log CFU/mL found, respectively, in the raw milk used for the Spanish cheeses Leòn (Rodriguez Medina et al., 1995) and Genestoso (Arenas et al., 2004; the latest was indeed considered an outlier).

Focusing on the LAB counts, cocci were found in very high concentrations at 37°C (5.88 log CFU/mL on average). However, this value is influenced by the high counts of raw milk used to produce artisanal cheeses from Sicily [7.9 log CFU/mL (Randazzo et al., 2002)], from Brasil [6.53 and 6.86 log CFU/mL in raw milk sampled in dry and rainy season, respectively, (Castro et al., 2016)], and from Turkey [6.64 log CFU/mL (Çetinkaya and Ece Soyutemiz, 2006)]. The raw milk used for artisanal cheese Caciotta di Montefeltro (Aquilanti et al., 2011) was also found to have a particular high value of mesophiles (22–25°C) coccoid LAB of 7.61 log CFU/mL. The same artisanal productions, together with raw milk used for artisanal Rugova cheese from Kosovo (Ajazi et al., 2018), Kashar from Turkey (Çetinkaya and Ece Soyutemiz, 2006), and Minas from Brazil (Castro et al., 2016), showed the highest count of lactobacilli at the same incubation temperature of 37°C, expanding the value range of LAB found in MRS (1st quartile = 3.69 log CFU/mL; 3rd quartile = 6.29 log CFU/mL), which had an average of 5.05 log CFU/mL (Figure 3). The concentration of lactobacilli able to grow at this temperature in raw milk used for Cheddar cheese was found to be 2.28 log CFU/mL (McSweeney et al., 1993), even if the authors performed the cultivation on a different growth medium named *Lactobacillus* Selection Agar (LBS, Baltimore Biological Laboratories, Rockville, MD, United States; Becton Dickinson Microbiology Systems, United States). The same medium incubated at 30°C resulted in a lactobacilli concentration of 1.94 log CFU/mL in the raw milk used for the same cheese type (Gelsomino et al., 2001; Hickey et al., 2007).

More common and thus more useful for comparison was the incubation of MRS and M17 at 30–32°C, performed in 33 (*n* = 82) and 26 (*n* = 70) studies respectively, and representing the raw milk used for 24 of the 42 types of cheese analyzed in the review. The average concentration of lactobacilli (3.69 ± 1.21 log CFU/mL) was found significantly lower (*p <* 0.001) than coccoid LAB (4.62 ± 1.07 log CFU/mL). This result is consistent with what we found in the meta-analysis of HTS studies on the raw milk microbiota, where *Lactococcus* and *Streptococcus* genera were present in greater abundance compared to *Lacticaseibacillus* and *Lactobacillus* genera.

At the same growth temperature, total acidifying mesophiles had similar counts in raw milk used for cheese in Savoie-Haute Savoie using Elliker medium (Michel et al., 2001). Few works instead quantified coccoid LAB at 22–25°C, thus the higher concentration found in comparison with counts at 30–32°C may not be relevant.

The thermophiles population was counted in raw milk used for Swiss-type (Beuvier et al., 1997), Fior di Latte di Agerola (Coppola et al., 2006), Caciotta di Montefeltro (Aquilanti et al., 2011), Piedmont hard cheese (Bautista-Gallego et al., 2014), Caciocavallo di Castelfranco (Giello et al., 2017), Caciocavallo Palermitano (Settanni et al., 2012), and PDO cheeses such as Saint-Nectair (Delbès et al., 2007), Montasio (Marino et al., 2008; Carraro et al., 2011), Provolone del Monaco (Aponte et al., 2008), Salers (Callon et al., 2004), Comté (Bouton et al., 1998) and Parmigiano Reggiano (Coppola et al., 2000; Gatti et al., 2008). Thermophilic streptococci were particularly high in raw milk used for Fior di Latte di Agerola (6.7 log CFU/mL on average), causing a wider data distribution in comparison with thermophilic lactobacilli, searched in the same cheeses, except for Saint-Nectaire, which accounted an average of 3.06 log CFU/mL.

With the intention to discriminate LAB present in raw milk that better adapt to the biochemical transformation of the substrate during cheesemaking, some authors have developed specific media: Whey Agar Medium (WAM; Gatti et al., 2003), Curd Agar Medium (CURDAM; Lazzi et al., 2007), Cheese Agar Medium (CAM; Neviani et al., 2009); Heterofermentative Isolini Agar (Isolini et al., 1990) and Arginine Bromocresol Meat Extract Vancomycin (ABEV; Sohier et al., 2012) where used instead to discriminate facultative and obligate heterofermentative lactobacilli, which are known to differently adapt and grow in cheese during ripening; finally, Cheese Ripening Bacteria Media (CRBM; Denis et al., 2001) was proposed to selectively count LAB growing on the cheese surface. Low concentration of thermophiles was found in raw milk used for Parmigiano Reggiano (Gatti et al., 2008; Neviani et al., 2009) and Grana Padano (Franciosi et al., 2011b, 2012; Monfredini et al., 2012) when cultivated on WAM. The raw milk used for the same cheeses showed a slightly higher concentration of LAB able to grow in curd and cheese media (Gatti et al., 2008; Neviani et al., 2009; Santarelli et al., 2013). Heterofermentative lactobacilli were mainly analyzed at 37°C using Isolini agar and ABEV, resulting in a mean concentration of 2.82 ± 0.98 log CFU/mL. Although these specific media were used by few studies for the quantification of LAB in raw milk, they have the potential to be applied for the selective recovery of subdominant LAB species often difficult to isolate (Neviani et al., 2009).

### 3.2. Other culture-dependent methods

An alternative method that enables the quantification of LAB in milk, based on their duplicative capacity, is the impedometric analysis. Similarly to plate count, this is a culture-dependent method, but differently, it is not based on the quantification of formed colony but on the detection and estimation of LAB metabolites during their growth.

Impedometric analysis measures the resistance that an alternating current finds when passing through a conductive culture medium, where microorganisms are developing (Yang and Bashir, 2008; Bancalari et al., 2016). When this method is applied to the study of LAB growth in milk, the system can quantify the presence of ions resulting from the degradation of lactose into lactic acid, between two electrodes (Lanzanova et al., 1993; Mucchetti et al., 1994).

Using an instrument such as the BacTrac 4300^®^, two impedance components can be revealed and measured over time. The first component is the overall relative change in conductivity compared to an initially recorded value (M%) and the second is the variation of the ionic double layer near the surface of the two electrodes (E%; Futschik and Pfutzner, 1995; Bancalari et al., 2019). Both measures are recorded every 10 min, and incubation time and temperature can be set depending on the chosen conditions.

By plotting these measures against time, conductimetric or impedometric curves can be obtained. If these curves are then fitted with the Gompertz equation, three different parameters can be obtained: Lag, Rate and Yend (Bancalari et al., 2016). Particularly, the first parameter, Lag, is measured in hours and is defined as the time that the LAB cells need to adapt to the analysis conditions, before starting to duplicate. This value is inversely correlated with the amount of LAB cells: the smaller the time, the higher the number of LAB cells (Bancalari et al., 2016).

However, even if both impedance analysis and plate counts depend on the physiological state of the cells and their concentration, the Lag value cannot be converted into CFU. In fact, the Lag value is a more complex concept that depends on several factors such as the LAB genus, the species, biotype and, definitely, the physiological state of LAB cells at the beginning of the analysis. As compared to plate count, this method allows to collect more information about the behaviors of LAB strains, and even small differences can be highlighted within the same species (Bancalari et al., 2016).

Taking into consideration all these aspects, the impedometric method was recently used to quantify the mesophilic LAB in raw milk used to produce Grana Padano by measuring M% every 10 min for 48 consecutive hours at 25°C (D’Incecco et al., 2020). The obtained results allow underlining differences between samples in Lag values, ranging from 15 to 20 h, that were attributed to the different initial concentrations of LAB cells (D’Incecco et al., 2020).

One of the most evident advantages of this technique applied to the analysis of raw milk is the possibility of increasing the throughput, by analyzing many samples and variables at the same time and easily having more than two repetitions of each sample, which makes subsequent statistical data analysis and interpretation more reliable, especially if compared to plate counts.

## 4. Conclusion and perspectives

The route of some LAB bearing positive features for cheese production, begins when they reach the raw milk. With the intention of defining which and how many LAB are present in cow raw milk used for cheese production, this review compared and discussed the results of the available studies that addressed this topic through culture-dependent and-independent methods.

The critical review of the literature highlighted an inconsistency in the definition of “raw milk,” a term that can be referred to milk sampled at the farm rather than from the cheese vat after skimming. Together with the sampling procedures, other factors such as different farming systems, milk pre-treatments, and seasonality, take part in the modulation of the raw milk microbiota.

The meta-analysis conducted with the use of FMBN was very effective to highlight the complexity of the microbiota of cow raw milk used for cheese production which is composed of over 45 phyla and showed how the genera belonging to LAB are not the most abundant in this microbial ecosystem. It also indicated how LAB belonging to *Lactobacillus* and *Lacticaseibacillus* genera are present in lower relative abundance in comparison with *Lactococcus* and *Streptococcus*.

This result is consistent when compared with LAB concentration evaluated by means of plate counts on agar media: lactobacilli were in fact found on average in lower concentration than coccoid LAB in the raw milk used for the production of 24 different cheese types.

This allows once more to conclude that both culture-dependent and-independent techniques complementarily describe the raw milk microbiota, and the combination of the two approaches restores a complete picture of which and how many LAB are present in the raw milk.

A better knowledge of both the amount and the species of LAB present in the raw milk could be useful in the perspective of monitoring and maintaining the biodiversity of the microbiome of this complex substrate through the managing of parameters at the farm and cheese making level and finally predict the expected outcomes in the resulting raw milk cheeses.

## Author contributions

LB and MG conceptualized the study. LB performed the investigation, data curation and visualization, and wrote the original draft. MG supervised the study. AL and MG contributed to the methodology of data elaboration and acquired the fundings. EB and MG cured the Section 3.2 of the manuscript. BB critically revised the paper. All authors contributed to manuscript revision, read, and approved the submitted version.

## Funding

This research has financially been supported by the Programme “FIL-Quota Incentivante” of University of Parma and co-sponsored by Fondazione Cariparma.

## Conflict of interest

The authors declare that the research was conducted in the absence of any commercial or financial relationships that could be construed as a potential conflict of interest.

## Publisher’s note

All claims expressed in this article are solely those of the authors and do not necessarily represent those of their affiliated organizations, or those of the publisher, the editors and the reviewers. Any product that may be evaluated in this article, or claim that may be made by its manufacturer, is not guaranteed or endorsed by the publisher.

## Footnotes

^1^ https://www.scopus.com/
