# Supplementary material for: Lactic acid bacteria in cow raw milk for cheese production: Which and how many?
Source: Front Microbiol. 2023 Jan 12;13:1092224. doi: 10.3389/fmicb.2022.1092224 (PMC9878191; doi:10.3389/fmicb.2022.1092224)
Supplement: Supplementary file 2 [file Data_Sheet_2.docx]

Supplementary Material

**Supplementary table 1**. Selection in the “Filter” section of FMBN version 4.1.2-Shiny App (De Filippis et al., 2018; Parente et al., 2019, 2016) to obtain the taxonomic studies object of the meta-analysis.

| **Field** | **Filter for:** |
| --- | --- |
| *Select one or more food codes* | *A02LY* |
| *sample type* | *sample* |
| *issues* | *from -1 to 0* |
| *nature* | *raw* |
| *process* | *none* |
| *spoilage* | *unspoiled* |

**Supplementary table 2**. List of taxonomic studies that analyzed cow raw milk samples used for cheese production through 16S rRNA gene amplicon high-throughput sequencing. FMBN = FoodMicrobionet (De Filippis et al., 2018; Parente et al., 2019, 2016); PDO = Protected Designation of Origin (EP and Council of EU, 2012)

| **FMNB study ID** | **Sample type** | **Raw-milk cheese** | **Reference** |
| --- | --- | --- | --- |
| ST7 | Cow milk, whole | Caciocavallo Pugliese | (De Pasquale et al., 2014) |
| ST8 | Cow milk, whole | Fontina PDO | (Dolci et al., 2014) |
| ST10 | Cow milk, whole | Caciocavallo Silano PDO | (De Filippis et al., 2016) |
| ST22 | Cow milk, whole | Caciotta | (Calasso et al., 2016) |
|  |  | Caciocavallo Pugliese |  |

**Supplementary table 2**. (continued).

| ST44 | Cow milk, whole | Caciocavallo di Castelfranco | (Giello et al., 2017) |
| --- | --- | --- | --- |
| ST74 | Cow milk, whole | Cheddar | (Falardeau et al., 2019) |
|  |  | Gruyere |  |
|  |  | Jarlsberg |  |
| ST107 | Cow milk, whole | Serra da Canastra | (Kamimura et al., 2020) |
| ST149 | Cow milk, whole | Grana Padano “Trentingrana” PDO | (Cremonesi et al., 2020) |
| ST178 | Cow milk, whole | Cheese South Tyrol area | (Nikoloudaki et al., 2021) |

**Supplementary table 3**. List of the 149 most abundant (weighted degree > 10) Operational Taxonomic Units identified in whole cow raw milk samples analysed in the studies of supplementary table 2, and visualized in the bipartite network (figure 2).

| **n** | **Operational Taxonomic Unit** | **Taxa level** | **Degree** | **Weighted degree** | **Weighted degree %** |
| --- | --- | --- | --- | --- | --- |
| 1 | *Pseudomonas* | genus | 246 | 5622.05 | 23.85 |
| 2 | *Lactococcus* | genus | 224 | 1706.73 | 7.24 |
| 3 | *Chryseobacterium* | genus | 172 | 1545.24 | 6.56 |
| 4 | *Acinetobacter* | genus | 241 | 1416.53 | 6.01 |
| 5 | *Ralstonia* | genus | 107 | 1341.35 | 5.69 |
| 6 | *Streptococcus* | genus | 211 | 847.62 | 3.60 |
| 7 | *Bacteroides* | genus | 163 | 826.56 | 3.51 |
| 8 | *Enhydrobacter* | genus | 161 | 776.76 | 3.30 |
| 9 | *Kocuria* | genus | 110 | 577.73 | 2.45 |
| 10 | *Lacticaseibacillus* | genus | 178 | 570.47 | 2.42 |
| 11 | *Lactobacillus* | genus | 157 | 554.82 | 2.35 |
| 12 | *Staphylococcus* | genus | 226 | 542.62 | 2.30 |

**Supplementary table 3**. (continued)

| 13 | *Leuconostoc* | genus | 167 | 272.20 | 1.15 |
| --- | --- | --- | --- | --- | --- |
| 14 | *Psychrobacter* | genus | 159 | 263.85 | 1.12 |
| 15 | *Macrococcus* | genus | 93 | 245.55 | 1.04 |
| 16 | *Burkholderia-Caballeronia-Paraburkholderia* | genus | 79 | 219.70 | 0.93 |
| 17 | *Veillonella* | genus | 77 | 206.53 | 0.88 |
| 18 | *Pantoea* | genus | 29 | 204.16 | 0.87 |
| 19 | *Paeniclostridium* | genus | 101 | 202.47 | 0.86 |
| 20 | *UCG-005* | genus | 149 | 198.00 | 0.84 |
| 21 | *Phascolarctobacterium* | genus | 124 | 176.82 | 0.75 |
| 22 | *Roseburia* | genus | 38 | 152.24 | 0.65 |
| 23 | *Bifidobacterium* | genus | 81 | 149.63 | 0.63 |
| 24 | *Rikenellaceae RC9 gut group* | genus | 156 | 149.58 | 0.63 |
| 25 | *Acidovorax* | genus | 78 | 142.66 | 0.61 |
| 26 | *Carnobacterium* | genus | 50 | 134.31 | 0.57 |
| 27 | *Serratia* | genus | 59 | 129.68 | 0.55 |
| 28 | *Lachnospiraceae* | family | 137 | 128.56 | 0.55 |
| 29 | *Alistipes* | genus | 162 | 126.20 | 0.54 |
| 30 | *Escherichia/Shigella* | genus | 95 | 122.43 | 0.52 |
| 31 | *Paracoccus* | genus | 89 | 122.18 | 0.52 |
| 32 | *Corynebacterium* | genus | 135 | 114.86 | 0.49 |
| 33 | *Jeotgalibaca* | genus | 116 | 113.30 | 0.48 |
| 34 | *Stenotrophomonas* | genus | 152 | 104.87 | 0.44 |
| 35 | *Halomonas* | genus | 93 | 101.43 | 0.43 |
| 36 | *Enterococcus* | genus | 157 | 96.30 | 0.41 |
| 37 | *UCG-010* | family | 126 | 84.97 | 0.36 |
| 38 | *Brevibacterium* | genus | 82 | 82.44 | 0.35 |

**Supplementary table 3**. (continued)

| 39 | *Flavobacterium* | genus | 156 | 81.05 | 0.34 |
| --- | --- | --- | --- | --- | --- |
| 40 | *Sphingomonadaceae* | family | 83 | 77.13 | 0.33 |
| 41 | *Caulobacter* | genus | 86 | 74.77 | 0.32 |
| 42 | *Shewanella* | genus | 68 | 74.04 | 0.31 |
| 43 | *Clostridium* | genus | 88 | 72.60 | 0.31 |
| 44 | *Propionibacterium* | genus | 33 | 63.90 | 0.27 |
| 45 | *Turicibacter* | genus | 143 | 61.47 | 0.26 |
| 46 | *Micrococcaceae* | family | 50 | 59.02 | 0.25 |
| 47 | *Christensenellaceae R-7 group* | genus | 120 | 59.00 | 0.25 |
| 48 | *Parabacteroides* | genus | 57 | 58.25 | 0.25 |
| 49 | *Atopostipes* | genus | 152 | 56.27 | 0.24 |
| 50 | *Prevotellaceae UCG-003* | genus | 126 | 55.03 | 0.23 |
| 51 | *Bacteria* | kingdom | 126 | 53.23 | 0.23 |
| 52 | *Bacteroidales RF16 group* | family | 142 | 52.19 | 0.22 |
| 53 | *Lechevalieria* | genus | 43 | 52.07 | 0.22 |
| 54 | *Prevotellaceae UCG-004* | genus | 107 | 51.15 | 0.22 |
| 55 | *Micrococcales* | class | 66 | 50.97 | 0.22 |
| 56 | *[Eubacterium] eligens group* | genus | 47 | 48.92 | 0.21 |
| 57 | *Weissella* | genus | 46 | 48.66 | 0.21 |
| 58 | *[Eubacterium] coprostanoligenes group* | family | 106 | 47.87 | 0.20 |
| 59 | *Bacillus* | genus | 70 | 47.11 | 0.20 |
| 60 | *Enterobacteriaceae* | family | 93 | 46.57 | 0.20 |
| 61 | *Prevotella* | genus | 107 | 45.84 | 0.19 |

**Supplementary table 3**. (continued)

| 62 | *[Ruminococcus] gnavus group* | genus | 23 | 45.68 | 0.19 |
| --- | --- | --- | --- | --- | --- |
| 63 | *Dermacoccus* | genus | 46 | 45.00 | 0.19 |
| 64 | *Actinobacteria* | class | 65 | 44.80 | 0.19 |
| 65 | *Enterobacter* | genus | 48 | 41.76 | 0.18 |
| 66 | *Aerococcus* | genus | 142 | 39.26 | 0.17 |
| 67 | *Solibacillus* | genus | 65 | 38.43 | 0.16 |
| 68 | *Cutibacterium* | genus | 103 | 36.18 | 0.15 |
| 69 | *Janthinobacterium* | genus | 44 | 36.15 | 0.15 |
| 70 | *Bacteroidales* | class | 129 | 35.95 | 0.15 |
| 71 | *Clostridiaceae* | family | 65 | 35.90 | 0.15 |
| 72 | *Monoglobus* | genus | 76 | 34.66 | 0.15 |
| 73 | *Ruminococcus* | genus | 91 | 32.72 | 0.14 |
| 74 | *Tepidiphilus* | genus | 16 | 32.64 | 0.14 |
| 75 | *Romboutsia* | genus | 40 | 29.99 | 0.13 |
| 76 | *Marinospirillum* | genus | 77 | 29.40 | 0.12 |
| 77 | *Caryophanon* | genus | 59 | 29.11 | 0.12 |
| 78 | *Microbacterium* | genus | 62 | 28.85 | 0.12 |
| 79 | *Sphingobacterium* | genus | 92 | 28.70 | 0.12 |
| 80 | *Latilactobacillus* | genus | 89 | 28.69 | 0.12 |
| 81 | *Massilia* | genus | 52 | 28.66 | 0.12 |
| 82 | *Bradyrhizobium* | genus | 63 | 27.94 | 0.12 |
| 83 | *Alphaproteobacteria* | class | 76 | 27.94 | 0.12 |
| 84 | *Allorhizobium-Neorhizobium-Pararhizobium-Rhizobium* | genus | 37 | 27.22 | 0.12 |
| 85 | *Blautia* | genus | 41 | 26.77 | 0.11 |
| 86 | *Paludibacteraceae* | family | 71 | 25.89 | 0.11 |
| 87 | *Microbacteriaceae* | family | 59 | 25.87 | 0.11 |

**Supplementary table 3**. (continued)

| 88 | *Aerococcaceae* | family | 75 | 23.80 | 0.10 |
| --- | --- | --- | --- | --- | --- |
| 89 | *Hafnia-Obesumbacterium* | genus | 30 | 23.67 | 0.10 |
| 90 | *Luteococcus* | genus | 41 | 23.53 | 0.10 |
| 91 | *Dietzia* | genus | 58 | 23.33 | 0.10 |
| 92 | *Ruminobacter* | genus | 57 | 23.09 | 0.10 |
| 93 | *WCHB1-41* | class | 85 | 23.04 | 0.10 |
| 94 | *p-2534-18B5 gut group* | family | 89 | 23.03 | 0.10 |
| 95 | *Comamonas* | genus | 51 | 22.98 | 0.10 |
| 96 | *Deinococcus* | genus | 52 | 22.81 | 0.10 |
| 97 | *Alloprevotella* | genus | 93 | 22.49 | 0.10 |
| 98 | *Oscillospiraceae* | family | 80 | 22.30 | 0.09 |
| 99 | *Facklamia* | genus | 102 | 22.19 | 0.09 |
| 100 | *Aeromonas* | genus | 22 | 21.70 | 0.09 |
| 101 | *Yersinia* | genus | 39 | 21.51 | 0.09 |
| 102 | *Comamonadaceae* | family | 99 | 21.30 | 0.09 |
| 103 | *Lentilactobacillus* | genus | 76 | 21.08 | 0.09 |
| 104 | *Muribaculaceae* | family | 89 | 21.06 | 0.09 |
| 105 | *Jeotgalicoccus* | genus | 130 | 20.53 | 0.09 |
| 106 | *Brucella* | genus | 50 | 19.32 | 0.08 |
| 107 | *Erysipelothrix* | genus | 68 | 18.81 | 0.08 |
| 108 | *Pedobacter* | genus | 87 | 18.72 | 0.08 |
| 109 | *dgA-11 gut group* | genus | 72 | 18.53 | 0.08 |
| 110 | *S15B-MN24* | class | 6 | 18.35 | 0.08 |
| 111 | *Paeniglutamicibacter* | genus | 32 | 17.03 | 0.07 |
| 112 | *Aeromicrobium* | genus | 23 | 16.73 | 0.07 |
| 113 | *Brachybacterium* | genus | 30 | 16.11 | 0.07 |
| 114 | *Treponema* | genus | 66 | 15.86 | 0.07 |

**Supplementary table 3**. (continued)

| 115 | *Pseudomonadaceae* | family | 64 | 15.78 | 0.07 |
| --- | --- | --- | --- | --- | --- |
| 116 | *Lelliottia* | genus | 34 | 15.64 | 0.07 |
| 117 | *Sphingomonas* | genus | 38 | 15.58 | 0.07 |
| 118 | *RF39* | class | 94 | 15.30 | 0.06 |
| 119 | *Alloiococcus* | genus | 62 | 15.19 | 0.06 |
| 120 | *Mailhella* | genus | 79 | 14.98 | 0.06 |
| 121 | *Nitrospira* | genus | 57 | 13.96 | 0.06 |
| 122 | *Afipia* | genus | 43 | 13.80 | 0.06 |
| 123 | *Lachnospiraceae NK3A20 group* | genus | 53 | 13.27 | 0.06 |
| 124 | *Dermabacteraceae* | family | 22 | 13.23 | 0.06 |
| 125 | *Haemophilus* | genus | 41 | 13.12 | 0.06 |
| 126 | *Mogibacterium* | genus | 77 | 13.09 | 0.06 |
| 127 | *Peptococcaceae* | family | 54 | 12.87 | 0.05 |
| 128 | *Hymenobacter* | genus | 32 | 12.84 | 0.05 |
| 129 | *Lachnospiraceae Incertae Sedis* | genus | 20 | 12.70 | 0.05 |
| 130 | Flavonifractor | genus | 21 | 12.56 | 0.05 |
| 131 | *Enterobacterales* | class | 49 | 12.43 | 0.05 |
| 132 | *Erwiniaceae* | family | 15 | 12.37 | 0.05 |
| 133 | *Carnobacteriaceae* | family | 113 | 12.20 | 0.05 |
| 134 | *Faecalibacterium* | genus | 52 | 11.80 | 0.05 |
| 135 | *Pseudoalteromonas* | genus | 41 | 11.46 | 0.05 |
| 136 | *Family XIII AD3011 group* | genus | 71 | 11.37 | 0.05 |
| 137 | *Citricoccus* | genus | 18 | 11.21 | 0.05 |
| 138 | *Ruminococcaceae* | family | 77 | 11.00 | 0.05 |
| 139 | *Mycobacterium* | genus | 49 | 10.87 | 0.05 |
| 140 | *Pelomonas* | genus | 60 | 10.74 | 0.05 |

**Supplementary table 3**. (continued)

| 141 | *Burkholderiales* | classe | 64 | 10.62 | 0.05 |
| --- | --- | --- | --- | --- | --- |
| 142 | *Brevundimonas* | genus | 47 | 10.50 | 0.04 |
| 143 | *Akkermansia* | genus | 54 | 10.40 | 0.04 |
| 144 | *Hungatella* | genus | 17 | 10.21 | 0.04 |
| 145 | *Rhodococcus* | genus | 23 | 10.20 | 0.04 |
| 146 | *Prevotellaceae UCG-001* | genus | 46 | 10.17 | 0.04 |
| 147 | *Aquabacterium* | genus | 55 | 10.15 | 0.04 |
| 148 | *Weeksellaceae* | family | 53 | 10.13 | 0.04 |
| 149 | *Proteobacteria* | phylum | 51 | 10.03 | 0.04 |

**References**

Calasso, M., Ercolini, D., Mancini, L., Stellato, G., Minervini, F., Di Cagno, R., De Angelis, M., Gobbetti, M., 2016. Relationships among house, rind and core microbiotas during manufacture of traditional Italian cheeses at the same dairy plant. Food Microbiology 54, 115–126. https://doi.org/10.1016/j.fm.2015.10.008

Cremonesi, P., Morandi, S., Ceccarani, C., Battelli, G., Castiglioni, B., Cologna, N., Goss, A., Severgnini, M., Mazzucchi, M., Partel, E., Tamburini, A., Zanini, L., Brasca, M., 2020. Raw Milk Microbiota Modifications as Affected by Chlorine Usage for Cleaning Procedures: The Trentingrana PDO Case. Frontiers in Microbiology 11, 1–14. https://doi.org/10.3389/fmicb.2020.564749

De Filippis, F., Genovese, A., Ferranti, P., Gilbert, J.A., Ercolini, D., 2016. Metatranscriptomics reveals temperature-driven functional changes in microbiome impacting cheese maturation rate. Scientific Reports 6, 21871. https://doi.org/10.1038/srep21871

De Filippis, F., Parente, E., Zotta, T., Ercolini, D., 2018. A comparison of bioinformatic approaches for 16S rRNA gene profiling of food bacterial microbiota. International Journal of Food Microbiology 265, 9–17. https://doi.org/10.1016/j.ijfoodmicro.2017.10.028

De Pasquale, I., Di Cagno, R., Buchin, S., De Angelis, M., Gobbetti, M., 2014. Microbial ecology dynamics reveal a succession in the core microbiota involved in the ripening of pasta filata Caciocavallo Pugliese cheese. Applied and Environmental Microbiology 80, 6243–6255. https://doi.org/10.1128/AEM.02097-14

Dolci, P., De Filippis, F., La Storia, A., Ercolini, D., Cocolin, L., 2014. rRNA-based monitoring of the microbiota involved in Fontina PDO cheese production in relation to different stages of cow lactation. International Journal of Food Microbiology 185, 127–135. https://doi.org/10.1016/j.ijfoodmicro.2014.05.021

EP, Council of EU, 2012. REGULATION (EU) No 1151/2012 OF THE EUROPEAN PARLIAMENT AND OF THE COUNCIL of 21 November 2012 on quality schemes for agricultural products and foodstuffs. Official Journal of the European Union L 343/1.

Falardeau, J., Keeney, K., Trmčić, A., Kitts, D., Wang, S., 2019. Farm-to-fork profiling of bacterial communities associated with an artisan cheese production facility. Food Microbiology 83, 48–58. https://doi.org/10.1016/j.fm.2019.04.002

Giello, M., La Storia, A., Masucci, F., Di Francia, A., Ercolini, D., Villani, F., 2017. Dynamics of bacterial communities during manufacture and ripening of traditional Caciocavallo of Castelfranco cheese in relation to cows’ feeding. Food Microbiology 63, 170–177. https://doi.org/10.1016/j.fm.2016.11.016

Kamimura, B.A., Cabral, L., Noronha, M.F., Baptista, R.C., Nascimento, H.M., Sant’Ana, A.S., 2020. Amplicon sequencing reveals the bacterial diversity in milk, dairy premises and Serra da Canastra artisanal cheeses produced by three different farms. Food Microbiology 89, 103453. https://doi.org/10.1016/j.fm.2020.103453

Nikoloudaki, O., Lemos Junior, W.J.F., Borruso, L., Campanaro, S., De Angelis, M., Vogel, R.F., Di Cagno, R., Gobbetti, M., 2021. How multiple farming conditions correlate with the composition of the raw cow’s milk lactic microbiome. Environmental Microbiology 23, 1702–1716. https://doi.org/10.1111/1462-2920.15407

Parente, E., Cocolin, L., De Filippis, F., Zotta, T., Ferrocino, I., O’Sullivan, O., Neviani, E., De Angelis, M., Cotter, P.D., Ercolini, D., 2016. FoodMicrobionet: A database for the visualisation and exploration of food bacterial communities based on network analysis. International Journal of Food Microbiology 219, 28–37. https://doi.org/10.1016/j.ijfoodmicro.2015.12.001

Parente, E., De Filippis, F., Ercolini, D., Ricciardi, A., Zotta, T., 2019. Advancing integration of data on food microbiome studies: FoodMicrobionet 3.1, a major upgrade of the FoodMicrobionet database. International Journal of Food Microbiology 305. https://doi.org/10.1016/j.ijfoodmicro.2019.108249
